# Are “Resting” Microglia More “M2”?

**DOI:** 10.3389/fimmu.2014.00594

**Published:** 2014-11-18

**Authors:** Jonathan D. Cherry, John A. Olschowka, M. Kerry O’Banion

**Affiliations:** ^1^Department of Pathology and Laboratory Medicine, University of Rochester School of Medicine and Dentistry, Rochester, NY, USA; ^2^Department of Neurobiology and Anatomy, University of Rochester School of Medicine and Dentistry, Rochester, NY, USA

**Keywords:** microglia, M2, alternative activation, neuroinflammation, resting, activation

Over the last decade, the concept and nomenclature of microglial phenotype polarization has been carried over from the peripheral macrophage literature. However, it is not entirely correct to view these two cell types as overlapping. Microglia, although related to macrophages, have several differences and their own unique repertoire of features. In particular, microglia arise from a distinct early yolk sac progenitor population and therefore have different developmental origins than macrophages (1). Furthermore, once in the central nervous system (CNS), microglia are maintained through local self-renewal (2). Under normal conditions, there is no infiltration of peripheral macrophages (3). Microglia also maintain expression profiles distinct from peripheral macrophages (4). This demonstrates that microglia are not simply macrophages that have migrated into the brain; rather, they are a distinct cell type.

These ideas have led to a reassessment of microglia activity, leading many researchers to shift their thinking on glial biology in general. One of the more outdated concepts carried over from macrophages is the idea that microglia in the healthy brain exist in a “resting” state. Through a variety of stimuli these “resting” cells can rapidly be “activated,” yielding microglia that are cytotoxic (5). These “activated” microglia were described in and were thought to play a major driving role in many neurodegenerative diseases (6). Thus, microglia “activation” took on a largely negative connotation. Taking cues from ongoing work in peripheral macrophages, this initial concept of microglia “activation” being generally harmful gave way to more specific ideas suggesting there was not just one kind of “activation.” Under the influence of either pro- or anti-inflammatory cytokines, microglia could be polarized into an inflammatory or anti-inflammatory phenotype, designated classically and alternatively activated microglia, respectively (7). Others have given different designations not solely based on inflammation but from inhibiting vs. healing functions, and label them M1 and M2, respectively (8). It is now becoming clear that the responses of microglia, like macrophages, are heterogeneous: these responses can include pro- and anti-inflammatory signatures within single cells and small, nearby populations and are driven by the local environment that can supply M1 and M2 polarizing cues simultaneously (9). Therefore, microglial responses are much richer than the dichotomous nomenclature suggests. It is common to represent microglia polarization as a spectrum with each respective phenotype occupying the extremes of the scale (10). However, it is unclear whether the diverse functional responses observed are a product of many different kinds of microglia subsets or simply varying ratios of M1 and M2 microglia. While there is much work that details this spectrum of activation, and much remains to be investigated, an interesting but overlooked area is the phenotype of the “resting” microglial cell.

Classically, those who study microglial function in a healthy, normal brain are not the same groups that study microglia in an “activated” or disease setting. However, it appears that the microglia in each of these settings may be more similar than originally thought. The notion that microglia are truly “resting” has long been cast aside. The advent of *in vivo* techniques, in particular 2-photon microscopy, has revealed the constant surveillance and activity of microglia, even in the absence of traditional activation signals (11). Therefore, it might be incorrect to view microglia in the normal, healthy CNS as a distinct population that can become “activated.” Rather, it seems likely that even at baseline, microglia are already on the activation spectrum. So the question arises, where on the spectrum are they?

Inflammation in the brain is typically associated with harmful outcomes. Even acute, low level inflammation can impair synaptic function, leading to cognitive dysfunction and behavioral abnormalities (12). Moreover, neuroinflammation has been recognized as a pathological hallmark in most chronic neurodegenerative diseases (13). This demonstrates the sensitivity of the brain to inflammation and the importance of protecting the CNS from insult. This protective role has generally been assigned to M2 microglia. Many investigators have observed protective effects of M2 cells; such as elevated neuronal survival and process extension after treatment with M2 conditioned media (14), or as reported in numerous papers that detail the beneficial effect of direct treatment with M2 inducing agents (15). However, all of these take place during pathology and do not consider the normal protective function of microglia. Thus, given the critical role of basal microglia in maintaining homeostasis, an attractive hypothesis is that under normal conditions, microglia are skewed toward a protective, anti-inflammatory phenotype. In fact many of the normal functions of microglia are reminiscent of M2 cells, although they are not as prominent as fully polarized cells. Even without stimulation, microglia are vital sources of important, neurosupportive cytokines such as IGF-1 and BDNF (16–18). Neuroprotective cytokine secretion is generally considered an M2 microglial function, but we now know it also occurs in “resting” microglia, albeit at lower levels. Indeed, in models that lack proper M2 inducing signals like IL-4^−/−^ and SCID mice, cognitive impairment is observed, which was attributed to decreased production of these necessary neurotrophins (19, 20).

Furthermore, microglia in a basal state share another important function with fully polarized M2 microglia, namely, rapid and efficient debris clearance (21). This process, which is one of the quintessential defining functions of M2 polarized cells, seems to be a default function for microglia. Although the concept of phagocytosis is not unique to alternatively activated microglia, the speed and quality at which this occurs differs between the phenotypes. In contrast to M1 polarized cells, where a slower and less acidic phagosome is beneficial for downstream immune functions such as antigen-presenting abilities, a rapid, more acidic phagosome aids M2 cells in quick, efficient removal of debris (22). This speed and efficiency can be observed in the highly dynamic process called synaptic pruning, which is characterized as rapid elimination of developing synapses. In the past several years, microglia have been shown to be crucial to this process (23). This concept is thought to be carried over into the adult but at a less dramatic level (24). The role of microglia in normal synaptic maintenance is not fully understood, so it is difficult to directly attribute baseline phagocytic function to an M2 related mechanism. However, due to the speed and efficiency at which this process occurs, we can speculate that this type of phagocytosis shares more similarities with M2 than M1 polarized microglia.

In addition to the neuroprotective and functional similarities resting microglia share with traditionally polarized M2 microglia, skewing to an M2 state can be seen in the receptor profile resting microglia express. For example, unlike macrophages, microglia express very low levels of MHCII (25). Only when microglia polarize to an inflammatory phenotype do they upregulate MHCII expression (26). Contrary to MHCII, DC-SIGN has been observed on microglia in the normal brain. This c-type lectin receptor, which has been implicated in promoting immune homeostasis, also maintains the immunosuppressive environment in the healthy brain (27). By limiting particular surface receptors while expressing others, microglia are biased toward a particular phenotype, namely anti-inflammatory. However, it appears that this M2 biased phenotype may change with age. For still unknown reasons, there is a loss of signals that keep microglia anti-inflammatory. During normal aging, a reduction in IL-4Rα is observed as well as a decreased sensitivity to other anti-inflammatory cytokines (28). This is mirrored by an increase in sensitivity to proinflammatory cytokines, suggesting a switch in the basal phenotype of microglia (29). A similar switch, termed priming, has been observed after microglia were exposed to inflammatory cytokines. Following the initial inflammatory insult, microglia appear to return to their basal state. However, with a second inflammatory stimulus these “primed” microglia produce significantly more inflammatory cytokines than unprimed microglia (30), suggesting that their basal state is altered toward a more M1 phenotype. Age-associated and priming switches may be involved in increased susceptibility to neurodegenerative disorders (31).

Keeping microglia skewed toward a non-inflamed state is critical for normal homeostasis and specific control mechanisms exist that actively prevent microglia from adopting an inflammatory profile. In particular, neurons express several receptors and ligands that signal to their counterparts specifically localized on microglia. CX3CR1, CD200, CD47, TREM2, and several other receptors have been identified that participate in constant cross-talk between microglia and neurons (32). Interestingly, in genetic knockout mice missing CX3CR1, impaired cognition was observed (33). Two explanations appear likely. First, the loss of direct inhibition resulted in increased inflammation, which in turn, caused cognitive dysfunction (33). Secondly, the loss of CX3CR1 resulted in impaired phagocytic ability (23) and subsequent loss of proper synaptic pruning during a critical developmental period, as discussed previously. Although different, both of these explanations share the idea that divergence from proper normal baseline function (namely M2 skewed functions) results in CNS pathology. Furthermore, this suggests that neurons, under normal conditions, are active in controlling microglial polarization. This environmental control on phenotype skewing poses an interesting question, is basal state polarization present in other tissue specific macrophages? The answer is most likely yes. However, it seems that other tissue macrophage cells such as peritoneal, lung, or splenic red pulp macrophages all exhibit greater diversity in their normal gene expression and most likely, basal function when compared to microglia, even though they a share common yolk sac progenitor (1, 34). Therefore, it is hard to directly compare between the cell types. This demonstrates the large role the environment plays in skewing cells to the needs of a specific tissue. In the CNS, an anti-inflammatory state is most beneficial so the environment favors slight M2 skewing. It would be interesting to characterize potential skewing in other organs and how that relates to normal resident macrophage function.

The idea that microglia exist as a skewed population in normal, non-pathological tissue highlights this phenotype as an innate characteristic of microglia. The classical view of macrophage biology is that the adaptive arm of the immune system, primarily T cells, controls phenotypes via release of cytokines such as IFN-γ or IL-4 (35, 36). Unfortunately, it is difficult to prove innate or adaptive control in peripheral tissue due to the normal presence of T cells. However, the healthy brain is largely devoid of T cells, which accentuates the idea that M2 phenotype skewing is a default, innate function of microglia (37). Interestingly, similar ideas have been proposed for macrophages (38).

In conclusion, to properly understand and discuss microglia, we have to do away with terms such as “activation” and “resting.” By using these outdated concepts, we fail to acknowledge the complex plastic nature of microglia. In addition to not correctly representing the non-pathological, normal functions of microglia, the term “activation” is vague and provides no specific information about the many possible microglial phenotypes. Microglia are always “active,” so the true distinguishing feature is where they exist on the phenotype spectrum. Healthy, normal brain microglia do not sit precisely in the center between inflammatory and anti-inflammatory cells. Rather, these sentinel microglia are slightly shifted toward an anti-inflammatory phenotype, which is beneficial to brain homeostasis. The field of microglial biology does not need to battle over nomenclature for baseline microglia. No single term can adequately encompass all microglial functions at all times. But it is important to recognize that these cells are a plastic population that can dynamically shift between a spectrum of phenotypes and should not be boxed into fixed, rigid “activation” states.

## Authors Contribution

Jonathan D. Cherry researched the literature and drafted the manuscript. M. Kerry O’Banion and John A. Olschowka critically reviewed and edited the work. All the authors read and approved the final manuscript.

## Conflict of Interest Statement

The authors declare that the research was conducted in the absence of any commercial or financial relationships that could be construed as a potential conflict of interest.
